# Female genital mutilation/cutting and obstetrical anal sphincter injury risk in the United States

**DOI:** 10.1016/j.xagr.2026.100657

**Published:** 2026-06-05

**Authors:** Emma Stirling-Cameron, Chelsey Perry, Jocelyn Stairs

**Affiliations:** 1 School of Population and Public Health, University of British Columbia, Vancouver, Canada (Stirling-Cameron and Perry); 2 Department of Obstetrics and Gynecology, Dalhousie University, Halifax, Canada (Stairs); 3 Department of Community Health & Epidemiology, Dalhousie University, Halifax, Canada (Stairs)

**Keywords:** episiotomy, female genital cutting, female genital mutilation, National Inpatient Sample, obstetrical anal sphincter injury, vaginal delivery

## Abstract

**BACKGROUND:**

Female genital mutilation/cutting (FGM-C) affects more than 230 million women and girls worldwide and carries increased risk of adverse obstetrical outcomes, including obstetrical anal sphincter injury (OASIS). With growing global migration, obstetrical care providers may increasingly encounter individuals affected by FGM-C in historically low-prevalence settings such as the United States. Therefore, it is essential to understand the association between FGM-C and adverse obstetrical outcomes in settings such as the United States to inform evidence-based practice.

**OBJECTIVE:**

To estimate the association between FGM-C and OASIS, among inpatient, singleton, vaginal deliveries among people delivering in-hospital in the United States. The secondary objectives were to estimate the association between FGM-C and postpartum hemorrhage and prolonged second stage of labor in this population and the role of episiotomy in mitigating risk of OASIS in this population.

**STUDY DESIGN:**

We conducted a population-based, retrospective cohort study of pregnant individuals delivering singleton fetuses in the United States using the Healthcare Cost and Utilization Project National Inpatient Sample (NIS) database (2016–2019, inclusive). Exposure and outcome variables were derived using International Classification of Diseases-10 disease and procedure codes. Weighted multivariate logistic regression models were used to estimate the odds ratio (OR) for the association between FGM-C and OASIS and secondary outcomes.

**RESULTS:**

During the study period, 2020,780 delivery-related discharges were captured, representing an estimated 10,168,193 singleton vaginal deliveries nationwide using the complex survey design of the NIS. A total of 795 (0.03%) individuals were diagnosed with FGM-C and 39,668 with OASIS (1.4%) during the study period. After adjusting for potential confounders, vaginal deliveries affected by FGM-C had 3.02 times the odds of sustaining OASIS (95% CI: 2.23–4.07), 1.99 times the odds of experiencing postpartum hemorrhage (95% CI: 1.45–2.73), and 2.33 times the odds of a prolonged second stage of labor (95% CI: 1.23–4.39) compared to unaffected individuals. Performance of episiotomy attenuated the risk of OASIS (aOR 1.12, 95% CI: 0.59–2.11).

**CONCLUSION:**

Singleton vaginal deliveries complicated by FGM-C have significantly increased odds of experiencing adverse obstetric outcomes, including OASIS, postpartum hemorrhage, and prolonged second stage of labor. Episiotomy may be protective against OASIS in this population.

**AJOG Global Reports at a Glance**

**Why was this study conducted?**

• Growing global migration increasingly requires providers in low-prevalence settings to be competent in the obstetrical management of individuals affected by female genital mutilation/cutting (FGM-C)
• To contribute to clinical guidance, specifically to estimate the association between FGM-C and obstetrical anal sphincter injury (OASIS) risk among singleton vaginal deliveries in the United States

**Key findings**

• We found that pregnant persons affected by FGM/C who underwent a vaginal delivery in the United States had increased odds of adverse intrapartum outcomes, including OASIS
• Episiotomy attenuated this risk

**What does this study add to what is known?**

• This study builds on prior studies on this topic by using a large, population-based database and examining a population of individuals delivering in the United States

## Introduction

Female genital mutilation/cutting (FGM-C) is estimated to affect over 230 million women worldwide across 30 countries in Africa, the Middle East and Asia.^1^ FGM-C refers to any procedure involving partial or complete removal of external female genitalia or other injury to the genital organs for nonmedical reasons.^2^ FGM-C is a cultural practice and is typically carried out on young females between infancy and the age of 15.^2^ FGM-C has no known health benefits and can cause severe bleeding, voiding dysfunction, labial cysts, bacterial vaginosis, and sexual dysfunction.^3^, ^4^, ^5^ The number of individuals in the United States at risk of FGM-C or its consequences tripled over a decade, with approximately 513,000 individuals at risk in 2012.^6^

FMG-C can contribute to obstetric complications during childbirth, including emergency cesarean section,^7^^,^^8^ episiotomy,^4^ postpartum infection,^7^ and perineal tearing.^3^^,^^4^ Emerging evidence suggests that FGM-C may increase the risk of obstetrical anal sphincter injury (OASIS),^8^^,^^9^ a condition with severe short- and long-term consequences, including urinary and anal incontinence, fistulae, sexual dysfunction, and psychological trauma.^10^^,^^11^ Existing studies on the association between FGM-C and OASIS are limited by study quality and sample size and rely heavily on descriptive estimates.^7^^,^^12^^,^^13^

The growing migration of individuals from regions where FGM-C is prevalent to North America highlights the need for healthcare systems in historically low-prevalence areas to develop expertise and guidance in managing individuals affected by FGM-C.^2^ Obstetrical management has been identified as a priority area for investigation, and American providers report feeling underprepared to care for individuals affected in this setting.^14^ Restrictive practice of mediolateral episiotomy is recommended more broadly as a strategy to minimize OASIS risk^15^; however, its role as a protective measure among individuals living with FGM-C and receiving care in the American healthcare system is unknown. Characterizing the risk of OASIS among birthing individuals with FGM-C in low-prevalence settings, such as the United States, can inform clinical best practice guidelines for reducing OASIS and future research identifying protective interventions in this population.

The primary objective of this study was to estimate the association between FGM-C and OASIS in a large, contemporary, nationally representative cohort of births in a low-prevalence setting. The secondary objectives of this study were to estimate the association between FGM-C and other adverse birth outcomes in this setting, including postpartum hemorrhage and prolonged second stage of labor, and to examine the role of episiotomy in OASIS risk.

## Materials and methods

### Study design

The Healthcare Cost and Utilization Project National Inpatient Sample (HCUP-NIS) was used to assemble a retrospective, population-based cohort of discharges between 2016 and 2019. These years were chosen to represent contemporary obstetrical practice while excluding the influence of the COVD-19 pandemic on care. This database leverages a large, nationally representative sample of inpatient deliveries, enabling efficient investigation of rare exposures like FGM-C. The NIS is a component of the HCUP sponsored by the Agency for Healthcare Research and Quality.^16^ The NIS includes a 20% stratified sample of discharges following inpatient hospital stays. It captures about 1000 hospitals from 47 US states, encompassing approximately 8 million hospital stays per year. It is the largest publicly available all-payer inpatient database in the United States.^17^

### Study population

Eligible participants included females discharged following admission to hospital for a singleton vaginal delivery between 2016 and 2019, inclusive. Pregnancies complicated by multiple gestation or cesarean section were not included. To identify singleton delivery admissions, we used a previously described approach to identify delivery admissions using the International Classification of Diseases (ICD) 10th edition.^17^ For the year 2019, we used the newly introduced “I10_BIRTH” variable to identify admissions where an in-hospital birth occurred.

### Exposure

The primary exposure variable was FGM-C. This was defined using ICD-10 codes, N90811 (type I: Partial or total removal or the clitoral glans and/or clitoral hood), N90812 (type II: Partial or total removal of the clitoral glans and the labia minora, with or without excision of the labia majora), N90813 (type III: Restriction of the vaginal opening, infibulation), and N90818 and N90810 (type IV: Encompasses all other procedures done to female genitalia for nonmedical purposes (eg, piercing, incising, cauterization).^18^ A binary variable (FGM-C yes/no) was used for the primary analysis. Descriptive analysis of FGM-C type was also included. Controls included individuals discharged following singleton deliveries who did not have FGM-C. This was defined by absence of these ICD-10 codes.

### Outcome

Obstetrical anal sphincter injury (OASIS) was our primary outcome of interest, defined using the Sultan classification as perineal laceration with damage to the anal sphincter and possibly anal mucosa, including third- and fourth-degree perineal lacerations. OASIS was defined by the following ICD-10 codes: O7O.20 (third degree perineal laceration, unspecified), O70.21 (type 3A: third degree perineal laceration), O70.22 (type 3B: third degree perineal laceration), O70.23 (type 3C: third degree perineal laceration), and O70.3 (fourth degree perineal laceration). A binary variable (OASIS yes/no) was used for the primary analysis. Descriptive analysis of grade of tear was also completed.

Secondary outcomes included postpartum hemorrhage and a delayed second stage of labor. Postpartum hemorrhage was defined as a cumulative blood loss of greater than or equal to 1000 mL or blood loss accompanied by signs of hypovolemia.^19^ Postpartum hemorrhage was defined using the following ICD-10 codes: O679, O721. A prolonged second stage of labor is defined in clinical guidance as more than 3 hours of pushing in nulliparous women, and 2 hours in multiparous women.^20^ This was defined using the following ICD-10 code: O631.

### Confounders and other covariates

Participant characteristics captured in the NIS datasets included maternal age, maternal race/ethnicity (defined as Black, non-Hispanic White, Hispanic, Asian and Pacific Islander, Native American, and unspecified), median income quartile by zip/area code ($1–$51,999, $52,000–$65,999, $66,000–$87,999, and $88,000+), and primary expected payer (Medicare, Medicaid, private, self-pay, no cost and other). Obstetric-related variables were defined using ICD-10 codes (Supplemental Appendix A). Additional obstetric characteristics considered to describe the population included large for gestational age infant, shoulder dystocia, gestational diabetes mellitus, pregnancy complicated by smoking, pregnancy complicated by obesity, pregnancy complicated by hypertension, pregnancy complicated by hypertensive disorder related to pregnancy, operative delivery (ie, forceps, vacuum), episiotomy, and preterm birth.

### Statistical analyses

Given that approximately 4.5% of pregnant persons are affected by OASIS,^21^ an estimated 680 deliveries of pregnancies affected by FGM/C would be required in order to detect an odds ratio (OR) of 1.60 for the primary exposure with 80% power, and alpha 0.05. We estimated a need to evaluate a total of 20,082 delivery admissions given that a prior retrospective cohort estimated an incidence of 3.2% of individuals in a population-based birth cohort were affected by FGM-C.^22^

Characteristics of the analytic sample were obtained using unweighted sample sizes and weighted population percentages, stratified by the exposure (FGM-C). Bivariate analyses were conducted using unadjusted linear regression for continuous variables (ie, age and length of stay) and Chi-squared and Fisher’s exact tests for categorical variables comparing demographic and obstetrics characteristics with exposure to FGM-C. Complete cases accounted for 95% of our analytic sample. Variables with missingness included primary expected payer (0.12% missing), income quartile by area/zip code (0.95% missing), and maternal race/ethnicity (4.28% missing).

Multivariable logistic regression with complete case analysis was used to estimate ORs with 95% confidence intervals to examine the association between FGM-C and OASIS, adjusting for the following prespecified potential confounders: age, race, shoulder dystocia, maternal obesity, and large for gestational age infant. Results are presented for the full cohort and stratified by performance of episiotomy and operative delivery, as prior study has suggested that episiotomy may be protective in women with FGM-C.^23^ For all multivariable analyses, survey procedures were used to account for the complex sampling design employed by the NIS database.^17^ All analyses were performed using R (The R Foundation, 4.4.2, 2024-10-31). Key packages used were *survey, srvyr, tableone, kableExtra*. All tests were two-tailed, and a *P* value of .05 was used to define statistical significance.

## Results

Demographic and obstetric characteristics are presented in Table 1, stratified by FGM-C. During the study period, 2020,780 delivery-related discharges were captured, representing an estimated 10,168,193 singleton deliveries nationwide (Figure). A total of 795 (0.03%) delivery admissions were for individuals affected by FGM-C, and 39,623 (1.4%) delivery admissions sustained an OASIS. Among deliveries affected by FGM-C, 63 (7.9%) were diagnosed with type 1107 (13.5%) type II, 150 (18.9%) type III, and 475 (59.8%) type IV. Most individuals with FGM-C were Black (80.7%). More than one-third (35.8%) of deliveries affected by FGM-C resided in an area code where the median income ranged from $1 to $51,999 (USD), and 520 (65.5%) had Medicaid as their primary insurer. Deliveries affected by FGM-C were significantly more likely to have received an episiotomy (22.5% vs 6.3%, *P*<.001), required an operative delivery (7.9% vs 5.6%, *P*=.006), and experience postpartum hemorrhage (7.5% vs 4.1%, *P*<.001).

**Table 1:** Baseline characteristics stratified by female genital mutilation/cutting, with unweighted sample size and weighted population percentage, among individuals discharged following singleton vaginal deliveries in the United States between 2016 and 2019

Variable	No FGM-C (*n*=2019,985)	FGM-C (*n*=795)	*P* value
**Age,***n* (%)	28.44 (5.87)	29.51 (5.15)	<.001
**Primary insurer,***n* (%)			<.001
Medicare	15,187 (0.8)	0 (0.0)	
Medicaid	869,639 (43.1)	520 (65.5)	
Private insurance	1019,922 (50.6)	239 (30.1)	
Self-pay	54,718 (2.7)	21 (2.6)	
No cost	1527 (0.1)	0 (0.0)	
Other	56,475 (2.8)	14 (1.8)	
**Length of stay** (mean, SD, d)	2.29 (1.68)	2.47 (2.22)	.003
**Race**, *n* (%)			<.001
White	1027,234 (53.2)	42 (6.0)	
Black	276,603 (14.3)	565 (80.7)	
Hispanic	402,255 (20.8)	NS	
Asian/Pacific Islander	120,522 (6.2)	12 (1.7)	
Native American	14,845 (0.8)	0 (0)	
Other	88,354 (4.6)	75 (10.7)	
**Income quartile,***n* (%)			<.001
$1–$51,999	560,347 (28.0)	285 (36.0)	
$52,000–$65,999	506,478 (25.3)	218 (27.6)	
$66,000–$87,999	499,388 (25.0)	184 (23.3)	
$88,000 or more	434,987 (21.7)	104 (13.1)	
**Obstetrical anal sphincter injury**, *n* (%)	39,623 (2.0)	45 (5.7)	<.001
**Perineal tear grade,***n* (%)			<.001
None	1422,623 (70.4)	453 (57.0)	
1	550,497 (27.3)	288 (36.2)	
2	39,201 (1.9)	41 (5.2)	
3 and 4	7664 (0.4)	13 (1.6)	
**Prolonged second stage**, *n* (%)	12,327 (0.6)	12 (1.5)	.002
**Large for gestational age,***n* (%)	29,891 (1.5)	15 (1.9)	.422
**Shoulder dystocia,***n* (%)	45,993 (2.3)	19 (2.4)	.925
**Gestational diabetes**, *n* (%)	10,197 (0.5)	NS	.808
**Smoker**, *n* (%)	101,894 (5.0)	0 (0)	<.001
**Obese**, *n* (%)	163,317 (8.1)	67 (8.4)	.772
**Essential hypertension,***n* (%)	39,806 (2.0)	NS	.019
**Hypertensive disorder of pregnancy**, *n* (%)	160,367 (7.9)	46 (5.8)	.029
**Operative delivery,***n* (%)	113,079 (5.6)	63 (7.9)	.006
**Preterm birth**, *n* (%)	74,960 (3.7)	11 (1.4)	.001
**Postpartum hemorrhage,***n* (%)	61,352 (4.1)	45 (7.5)	<.001
**Episiotomy,***n* (%)	127,699 (6.3)	179 (22.5)	<.001

Data are mean ± standard error or *n* (%). Number of missing values: race, 130; income by quartile, 6; primary expected payer, 2. NS: *n* < 10 is not reported for confidentiality purposes.

*FGM-C*, female genital mutilation/cutting; *OASIS*, obstetric anal sphincter injury.

**Figure:**
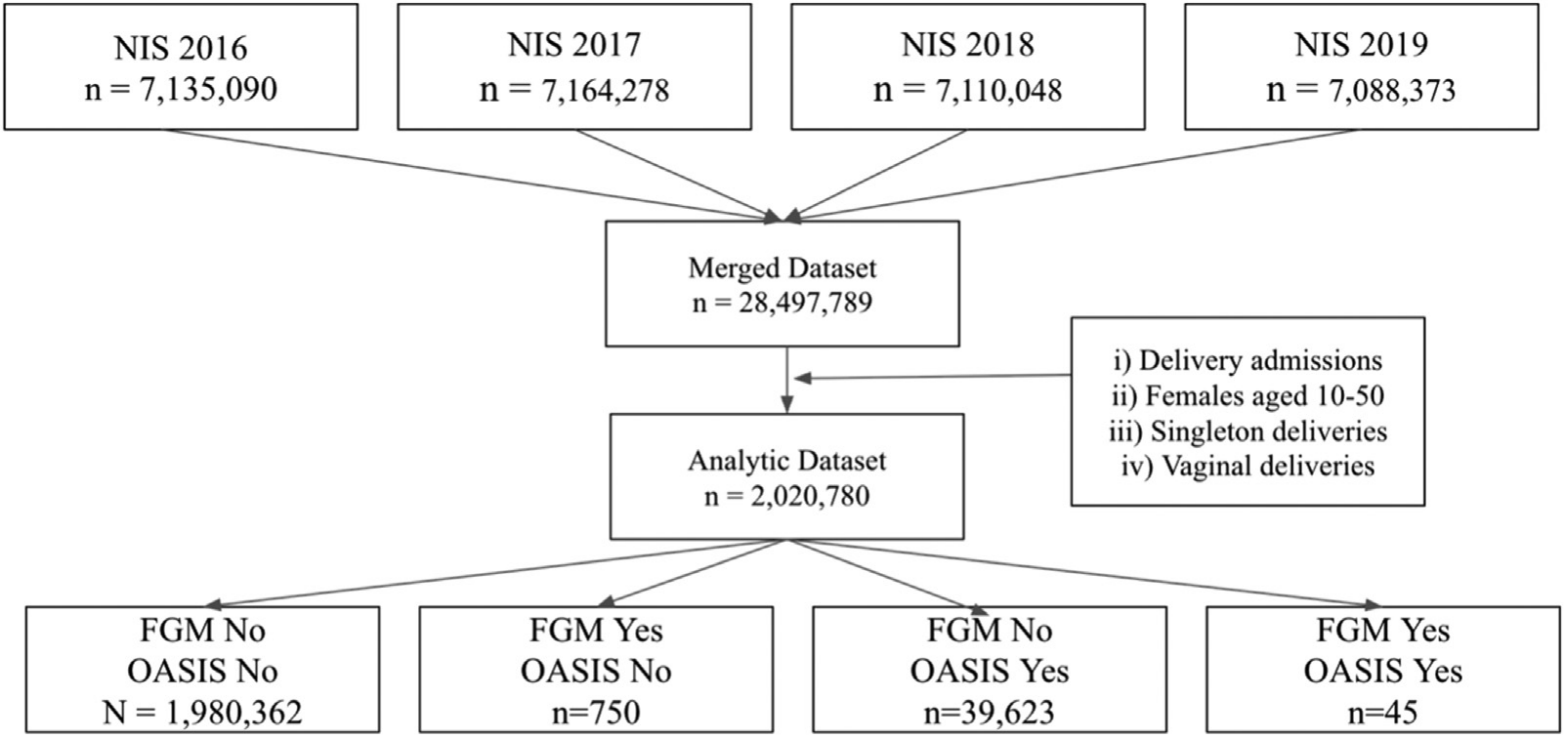
Flowchart of analytic sample extracted from 2016, 2017, 2018, and 2019 National Inpatient Sample (NIS) cycles to investigate the primary association between female genital mutilation/cutting (FGM-C) and obstetrical anal sphincter injury (OASIS).

Stirling-Cameron. Female genital mutilation/cutting and obstetrical anal sphincter injury risk. AJOG Glob Rep 2026.

After adjusting for age, race, maternal obesity, shoulder dystocia, large for gestational age infants, vaginal deliveries affected by FGM-C had significantly greater odds of experiencing OASIS compared to deliveries not affected by FGM-C (aOR 3.02, 95% CI: 2.23–4.07; Table 2). Additionally, in adjusted analyses, deliveries affected by FGM-C had significantly greater odds of experiencing postpartum hemorrhage (OR 1.99, 95% CI: 1.45–2.73) and a prolonged second stage of labor (OR 2.33, 95% CI: 1.23–4.39), when compared to deliveries not affected by FGM-C in unadjusted analyses (Table 3). Effect estimates remained constant and statistically significant in adjusted multivariable analyses.

**Table 2:** Association between female genital mutilation/cutting (FGM-C) and adverse obstetric outcomes among individuals discharged following singleton vaginal deliveries in the United States between 2016 and 2019

	OASIS	Postpartum hemorrhage	Prolonged second stage of labor
Crude OR (95% CI)	3.00 (2.27–3.96)^d^	1.90 (1.39–2.60)^d^	2.50 (1.41–4.42)^d^
Adjusted OR (95% CI)	3.02 (2.23–4.07)^a^^,^^d^	1.99 (1.45–2.73)^b^^,^^d^	2.33 (1.23–4.39)^c^^,^^d^

*CI*, confidence interval; *OR*, odds ratio.

a Model adjusted for age, race, maternal obesity, shoulder dystocia, large for gestational age infants.

b Model adjusted for adjusted for age, large for gestational age infant, shoulder dystocia, prolonged second stage, and hypertensive disorder of pregnancy.

c Model adjusted for adjusted for age, large for gestational age infant, and shoulder dystocia.

d *P*<.001.

**Table 3:** Association between female genital mutilation/cutting (FGM-C) and obstetric and anal sphincter injury (OASIS) stratified by episiotomy status, among individuals discharged following singleton vaginal deliveries in the United States between 2016 and 2019

Stratum	Model	Odds ratio, (95% CI)
No episiotomy	Crude	3.55 (2.55–4.94)
	Adjusted^a^	3.34 (2.31–4.81)
Episiotomy	Crude	0.88 (0.47–1.66)
	Adjusted^a^	1.12 (0.59–2.11)

*CI*, confidence interval.

a Model adjusted for age, race, maternal obesity, shoulder dystocia, and large for gestational age infants.

Among delivery admissions where an episiotomy was not performed, FGM-C was associated with significantly increased odds of OASIS, with an adjusted OR of 3.55 (95% CI: 2.55–4.94) compared to unaffected deliveries. In contrast, no significant association was observed between FGM-C and OASIS among individuals who had an episiotomy, with an adjusted OR of 1.12 (95% CI: 0.59–2.11).

## Discussion

### Principal findings

We investigated the association between FGM-C and adverse intrapartum outcomes in a population-based cohort of more than two million discharges following singleton vaginal delivery in the United States. Among deliveries affected by FGM-C, individuals faced three-times greater odds of experiencing OASIS and approximately two-times greater odds of experiencing prolonged second stage of delivery and postpartum hemorrhage when compared to deliveries unaffected by FGM-C. The association between FGM-C and OASIS was attenuated in deliveries where an episiotomy was performed.

### Results

Our findings align with prior studies that have documented adverse obstetric outcomes at the time of vaginal delivery among individuals with FGM-C.^9^^,^^24^, ^25^, ^26^ Eshraghi et al^9^ found a similar effect estimate using data from the Swedish Medical Birth Register (2014–2018); primiparous, singleton deliveries affected by FGM-C faced increased odds of experiencing OASIS, after adjusting for age, BMI, birthweight, instrumental delivery and episiotomy. Our study builds on these findings by considering episiotomy as an effect modifier rather than confounder, and our findings suggest a protective effect. Bonavina et al^25^ conducted a small prospective cohort study (*n*=345) in Sudan, a higher prevalence setting, that similarly found uncomplicated singleton deliveries affected by type III FGM-C faced greater odds of experiencing third or fourth degree perineal lacerations, after adjusting for maternal age and parity (aOR 2.28, 95% CI: 0.42–12.38). This study also similarly found that individuals affected by type three FGM-C faced increased odds of experiencing a prolonged second stage of labor and postpartum hemorrhage.^25^ Our findings build on those of Bonavina et al^25^ by demonstrating an association between FGM-C and OASIS that is sustained in a broader cohort of women with all four types of FGM-C.

In this study, we estimated that 0.03% of deliveries during the study period were affected by FGM-C. Prevalence estimates of FGM-C, especially in low-prevalence settings, remain limited and are largely based on population and migration modelling. The estimate in our birth cohort was lower than that of an Austrian birth cohort, in which an estimated 3.2% of birthing individuals were affected.^22^ This may reflect different migration patterns and population composition between the United States and Austria. It could also reflect the known limited training in FGM-C recognition and care among US healthcare providers,^14^ which may limit recognition of individuals affected by FGM-C at the time of delivery admission.

### Clinical implications

We hypothesize that the increased risk of OASIS in individuals affected by FGM-C has been secondary to decreased elasticity of the scarred labial tissue, which has previously been described.^26^ Consistent with our findings, episiotomy has previously shown to be protective against OASIS among individuals affected by FGM-C.^23^ However, in higher prevalence settings, other types of episiotomies such as anterior episiotomies may be performed,^23^ limiting the generalizability of these findings to settings like the United States where only mediolateral episiotomy would be encountered. It is likely that episiotomies, like antepartum defibulation, increase the introital aperture and may direct the tear away from the anal sphincter complex.

### Research implications

Future study clarifying the type of episiotomy and its protective effect in different subpopulations of individuals affected by FGM-C will aid in developing guidance around recommendations regarding when to perform episiotomy in this population. In the interim, our findings suggest that performance of an episiotomy may attenuate the increased OASIS risk observed in this population and should be considered.

### Strengths and limitations

To our knowledge, this is the first study examining the association between FGM-C and OASIS in an American healthcare setting. This study utilizes a large, nationally representative sample derived from a database with previously accrued validation evidence to estimate the association between FGM-C and OASIS. The large cohort size allows for more precise estimates and estimation of association involving a rare exposure (FGM-C) in a low-prevalence country. Estimations of risk in the United States contextualize these findings within contemporary American obstetrical practice, which improves their relevance to inform practice change. Further, use of a database that captures a representative sample of hospital types and geographic locations means that these findings are likely generalizable to patients admitted for singleton delivery admission in the United States. Finally, we used published models to promote accurate identification of delivery admissions using ICD-10 codes.^18^

The main limitation of this study, inherent to all retrospective, administrative database studies, is the potential for unaccounted for differences between groups, which could contribute to residual confounding. One potential confounder that has previously been associated with adverse health outcomes in individuals from countries with high prevalences of FGM/C is racism.^27^ Given the known association between race and/or ethnicity and adverse obstetrical outcomes, this will be an important component of future study. Further, we were unable to ascertain parity from the HCUP-NIS database. However, we anticipate that if any between-group differences exist, we would expect a larger number of multiparous patients in the exposure group, given higher reported intended family size among individuals from sub-Saharan African countries compared to the United States.^28^ Given existing knowledge about OASIS risk, we would expect that this would dampen the effect in the exposed group and therefore inclusion would be expected to strengthen the association. Additionally, there is the possibility of misclassification of secondary to missing ICD codes, despite the quality control procedures associated with the HCUP-NIS database.^16^ However, the group affected by FGM-C is likely truly exposed, and that the misclassified group is small compared to the total number of delivery admissions.

### Conclusions

Individuals affected by FGM-C had an increased odds of OASIS at the time of singleton vaginal delivery compared to unaffected individuals in a nationally-representative American cohort. Interventions such as episiotomy may mitigate risk of OASIS in this population.

## CRediT authorship contribution statement

**Emma Stirling-Cameron:** Writing – original draft, Methodology, Formal analysis, Conceptualization. **Chelsey Perry:** Writing – original draft, Methodology. **Jocelyn Stairs:** Writing – review & editing, Writing – original draft, Supervision, Funding acquisition, Formal analysis, Conceptualization.

## Appendix Supplementary materials

Image, application 1

## Footnotes

Supplementary material associated with this article can be found in the online version at doi:10.1016/j.xagr.2026.100657.
