# Supplementary material for: Female genital mutilation/cutting and obstetrical anal sphincter injury risk in the United States
Source: AJOG Glob Rep. 2026 Jun 5;6(3):100657. doi: 10.1016/j.xagr.2026.100657 (PMC13319998; doi:10.1016/j.xagr.2026.100657)
Supplement: Supplementary file 1 [file mmc1.docx]

**Supplementary Table 1**. Obstetric characteristics for study covariates and corresponding ICD-10 codes

| **Variable** | **ICD-10 Code** |
| --- | --- |
| Prolonged second stage | O631, Z3A |
| Postpartum hemorrhage | O72X |
| Infant large for gestation age | O3660X0, O3661X0, O3662X0, O3663X0 |
| Shoulder dystocia | O660 |
| Gestational diabetes mellitus | O2431, Z7985, Z794, O24311, O24.312, O24.313, O24.314, O24.315, O24.316 |
| Pregnancy complicated by smoking | O99.330, O99.331, O99.332, O99.333, O99.334, O99.335 |
| Pregnancy complicated by maternal hypertension | O10, I10_DX |
| Pregnancy complicated by hypertensive disorder related to pregnancy | O134, O114, O151 |
| Pregnancy complicated by obesity | O99.21, O99.210, O99.211, O99.212, O99.213, O99.214, O99.215 |
| Operative delivery (i.e., forceps, vacuum) | O81.0, 10D07Z3, 10D07Z4, 10D07Z5, O81.1, O81.2, O81.3, O81.4, 10D07Z6, O81.5, O66.5 |
| Episiotomy | 0W8NXZZ |
| Preterm birth | O6010X0, O6012X0, O6013X0, O6014X0 |
